# Clinical Trials and Novel Pathogens: Lessons Learned from SARS

**DOI:** 10.3201/eid1003.030702

**Published:** 2004-03

**Authors:** Matthew P. Muller, Allison McGeer, Sharon E. Straus, Laura Hawryluck, Wayne L. Gold

**Affiliations:** * Mount Sinai Hospital, Toronto, Ontario, Canada; † University Health Network, Toronto, Ontario, Canada; ‡ University of Toronto, Toronto, Canada

**Keywords:** Severe Acute Respiratory Syndrome, randomized controlled trials, disease outbreaks

## Abstract

During the recent global outbreak of severe acute respiratory syndrome (SARS), thousands of patients received treatments of uncertain efficacy and known toxicity such as ribavirin and corticosteroids. Despite this, no controlled clinical trials assessing the efficacy of these agents were conducted. If a second global SARS outbreak occurred, clinicians would not have controlled data on which to base therapeutic decisions. We discuss the unique methodologic and logistical challenges faced by researchers who attempt to conduct controlled trials of therapeutic agents during an outbreak of a novel or unknown infectious pathogen. We draw upon our own experience in attempting to conduct a randomized controlled trial (trial) of ribavirin therapy for SARS and discuss the lessons learned. Strategies to facilitate future clinical trials during outbreaks of unknown or novel pathogens are also presented.

The recognition of SARS as a transmissible disease prompted international efforts to identify its cause and control its spread. The success of these efforts has been dramatic, with the identification of the SARS-associated coronavirus (SARS-CoV) and the control of SARS outbreaks in all affected countries (*1*–*4*). An evidence-based approach to the management of the patient with SARS is still lacking, however, as no controlled clinical data are available to justify any of the treatments used. If SARS reemerges, clinicians will have little evidence on which to base treatment decisions. Could clinical trials have been conducted during the global outbreak? If so, what steps need to be taken to ensure that such trials are implemented appropriately the next time a similar event occurs?

We highlight the challenges faced by researchers attempting to conduct clinical trials of therapeutic agents during an outbreak caused by an unknown or novel pathogen. We focus the discussion on the design and implementation of randomized trials of candidate therapeutic agents, as trials are the gold standard on which therapeutic decision-making should be based. Examples from our own experience attempting to launch a trial of ribavirin therapy for SARS will illustrate these challenges.

## Formulating the Research Question

The first step in conducting a clinical trial is to develop a simple, testable hypothesis. The challenge facing researchers at the beginning of an outbreak caused by an unknown or novel pathogen is selecting a hypothesis in the face of scarce but rapidly evolving information. Once a hypothesis is selected and a trial started, new information arising during the outbreak may undermine the study hypothesis before the trial is completed.

In the initial phases of the SARS outbreak, empiric therapy was used to provide coverage against a broad differential of bacterial and viral pathogens. Ribavirin was included for coverage of a presumptive viral illness, given its in vitro activity against a variety of RNA and DNA viruses (*5*). As data on pathogenesis accrued, a hypothesis suggesting that lung injury may be immune mediated led to widespread use of corticosteroids in combination with ribavirin (*6*,*7*). Finally, isolation of SARS-CoV allowed for in vitro susceptibility studies that, in combination with increasing reports of toxicity attributed to ribavirin, resulted in discontinuation of ribavirin as a treatment for SARS in Toronto, although not in all affected areas (*8*,*9*).

Presently, a trial of corticosteroids appears to be a rational direction for future treatment trials, given their widespread use in the treatment of SARS, despite minimal supportive evidence, and their potential risks. Early in the outbreak, however, we focused our attention on designing a trial to evaluate the efficacy of ribavirin, which was being used in Canada and Hong Kong and had been associated with clinical successes in uncontrolled reports (*10*,*11*). We believed that conducting such a trial was important, given the widespread use of ribavirin and its potential risks and benefits. Subsequently, reports of adverse events associated with its use, demonstrated lack of in vitro activity, and reports of clinical progression on therapy convinced Canadian clinicians to discontinue its use without further study (*8*,*9*,*12*). At this point, we do not feel that a trial of ribavirin therapy is warranted. Some may argue that such a trial would have been valuable, given the continued use of ribavirin for the treatment of SARS in Hong Kong and China (*13*).

The hypothesis selected for evaluation during an outbreak must address an important clinical question than cannot be rapidly answered by other means. Although there may be a temptation to launch a trial rapidly after the onset of an outbreak, in most cases the etiologic agent should be identified with reasonable certainty before conducting trials of specific agents. Agents selected for study should have plausible mechanisms of action, activity established by in vitro studies, known toxicity profiles, and preliminary clinical evidence supporting their efficacy. Evidence should not consist solely of anecdotal case reports but should include some data derived from comparisons of treated and untreated patients. If other means of rejecting the hypothesis exist (i.e., in vitro studies, animal studies), but have not yet been conducted, a trial of the agent should be deferred and its resources allocated to trials testing agents that cannot be evaluated by other means. For example, the hypothesis that steroids can improve outcome in SARS by preventing or abrogating immune-mediated lung injury will be difficult to disprove by any means short of a trial.

If a trial is not considered justified, feasible, or ethical, therapeutic agents of unknown efficacy should not be used without attempts to systematically document illness severity, clinical course, treatment, adverse events, and confounding factors in a standardized manner to facilitate analysis. At a minimum, comparisons of cohorts of patients treated differently may yield information that can inform subsequent trials.

## Identifying the Study Population

In all clinical trials, the patient population to be included must be precisely defined. In an outbreak involving an unknown or novel pathogen, specific microbiologic tests to diagnose infection will not be available. Case definitions used by public health authorities to identify patients and to limit transmission through isolation and quarantine are deliberately broad to ensure that few contagious persons are missed. As a result, the specificity of diagnosis may be poor. Conducting a trial without a specific diagnostic test may result in the inclusion of patients without the condition of interest. These patients will receive potentially toxic therapies with no possible benefit. Furthermore, inclusion of patients without the disease of interest will bias the study towards the null hypothesis.

The initial case definitions for SARS are examples of this problem. Based on the World Health Organization’s initial case definition, persons presenting with fever, respiratory symptoms, and an epidemiologically defined exposure are considered to have SARS (*14*). This definition is non-specific, as all patients with febrile respiratory illnesses, regardless of etiology, will be included if they have any exposure history. This was particularly problematic in returned travelers with respiratory illness. Because direct SARS exposure was uncommon among travelers, even to high-risk areas, inclusion of travel-related cases in our trial would have resulted in a low positive predictive value for the case definition.

Although no simple solution to this problem exists, the specificity of inclusion criteria may be improved by ensuring that appropriate microbiologic investigations are performed to exclude patients with alternative diagnoses and by narrowing the epidemiologic case definition. For example, in the case of SARS, limiting study inclusion to those with pulmonary infiltrates on chest radiograph and requiring that epidemiologic links be based on direct contact with another case of SARS (which would exclude the majority of travelers to SARS-affected regions) would improve specificity. However, one must be careful when specificity is increased by selecting patients with more advanced presentations of disease because these patients may be less likely to respond to therapy compared with patients identified earlier in their disease.

Finally, post-hoc analysis of trial results can be performed once specific microbiologic and serologic tests become available. This analysis will allow further assessment of therapeutic efficacy. However, every effort should be made during the trial to exclude patients with a low probability of having the disease of interest to minimize the likelihood of treating uninfected patients with potentially toxic agents. This will minimize the number of patients treated inappropriately and maximize the likelihood of identifying differences in outcomes between the treatment and control groups.

## Defining the Intervention

All clinical trials require that the intervention used be defined explicitly. Dose, frequency, duration, and route of administration must be predetermined and are usually based on data from in vitro, animal, human safety, and dose-finding studies. In an outbreak involving an unknown or novel pathogen, these data will be unavailable. Choosing an intervention will be based on analogy to the treatment of other infectious diseases and available safety data. Negative trial results will be open to the criticism that a different dose or duration of therapy might have been effective.

In Toronto, the initial dose of ribavirin used was based on limited clinical experience with the use of high-dose ribavirin in the treatment of hemorrhagic fever syndromes (*15*). A similar dose was selected for our trial. Clinicians in Toronto who treated patients with SARS observed a high incidence of adverse effects associated with this regimen (*9*,*12*). As a result, regimens using a lower dose emerged, and ribavirin therapy was ultimately abandoned. Had our initial trial been launched, clinicians would have been unwilling to enroll patients, due to the high doses of ribavirin proposed and the emerging reports of drug toxicity.

Ideally, interventions selected for a trial should be based on preliminary data. Outbreaks of unknown and novel pathogens represent unique situations in which such data will not be available. The initial use of supportive care alone is a defendable position, particularly when minimal evidence exists to support the use of any therapeutic agent, and considerable evidence exists to support potential harm. This approach was adopted in the United States, and no fatal cases were reported, although only eight U.S. cases have been serologically confirmed (*4*). In parts of the world where outbreaks were larger, considerable pressure was placed on clinicians to offer specific therapies directed against SARS-CoV, even if such therapies were unproven and potentially dangerous. When unproven and potentially harmful therapeutic agents are being used in clinical practice, a simple, rapidly conducted trial may be beneficial. Protocols will need to allow for the evolution of clinical practice during the outbreak. Although changes in dose or duration may make interpretation of results more difficult, rigid adherence to initial protocols may make trials unacceptable to clinicians and potentially dangerous to patients. Such trials require ongoing communication with trial participants and timely disclosure of emerging information about efficacy and safety.

## Defining Study Outcomes

All clinical research requires a clearly defined and clinically relevant outcome. In an outbreak caused by an unknown or novel pathogen, the ultimate size of the outbreak will dictate enrollment and cannot be predicted in advance. Concerns that the trial will be underpowered are likely to arise. To enhance feasibility, selecting more frequently occurring, but less clinically relevant, outcomes may be tempting.

Estimates of case-fatality rates for the Toronto SARS outbreaks varied from 30%, to 3%, to 6.5%, and then to 17% as the outbreak progressed (*9*,*16*,*17*). Our initial sample size calculations using mortality as the outcome were based on a case-fatality estimate of 3% in the treatment (ribavirin) group. Using an alpha of 0.05 and a power of 80%, the sample size required to detect a 25% reduction in case-fatality rate from 4% in the placebo group to 3% in the ribavirin group would be 191 patients per group**.** Even if every SARS patient in Canada had been successfully enrolled, the study would not have recruited sufficient patients. Our solution was to use a composite outcome of death, mechanical ventilation, or severe hypoxemia to reduce the required sample size.

Due to the unknown size of an outbreak, the use of composite endpoints is a reasonable strategy to maximize the number of observed outcomes and minimize the required sample size, provided every component of the composite endpoint is clinically relevant and can be measured in a standardized manner.

## Challenges to Recruitment

The feasibility of conducting a trial during an outbreak is dependent on the ability to recruit sufficient numbers of patients into the study before the outbreak ends. In the first Toronto outbreak, SARS developed in 249 patients between the time the outbreak was recognized on March 13, 2003, and the end of the outbreak on April 25 (Figure). The decision to conduct a trial was made on March 23, 10 days after the recognition of the first cases of SARS. A protocol was rapidly developed and approved by the first local research ethics board on March 28 and by Health Canada on March 31. Review of the epidemic curve shows that the 18-day delay from the time the outbreak was recognized on March 13 to the receipt of expedited ethics approval on March 31 resulted in loss of 149 (60%) of 249 potential patients who could otherwise have been assessed for enrollment (Table). Cases that occurred during the second Toronto outbreak were not considered, as the use of ribavirin had been rejected by clinicians. Even if the second outbreak was considered, few additional case-patients could have been recruited because increased knowledge about control strategies resulted in reduced transmission and a smaller outbreak.

**Figure:**
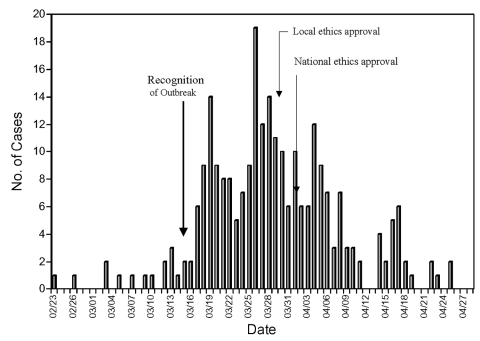
Epidemic curve of the first Toronto SARS outbreak. Data provided courtesy of the Ministry of Health and Long Term Care, Ontario, Canada.

**Table:** Eligible cases lost to enrollment in a hypothetical trial of ribavirin during the first outbreak of SARS in Toronto.

Date from outbreak recognition to date of enrollment of first patient (days)	Number of cases lost to enrollment (%)
March 13–16 (3)	6/249 (2)
March 13–20 (7)	37/249 (15)
March 13–23 (10)	62/249 (25)
March 13–28 (15)^a^	114/249 (46)
March 13–31 (18)^b^	149/249 (60)

^a^Approval from first local ethics review board ^b^Approval from Health Canada

The ability to launch a trial quickly in the face of an outbreak is dependent on the speed of protocol development and the time required for obtaining ethics approval and study funding. Strategies that could be developed before the next outbreak of an unknown or novel pathogen to facilitate the rapid initiation of trials include the establishment of a collaborative multicenter research network, the creation of a contingency fund for urgent therapeutic trials, and the development of new processes for emergency expedited ethics review.

## Value of a Collaborative Research Network

A national or international collaborative research network consisting of infectious disease clinicians, microbiologists, epidemiologists, and clinical trial methodologists should be established. The network would facilitate the rapid initiation of trials during an outbreak through the advanced preparation of study protocols and by providing urgent assistance in the design and implementation of trials after recognition of an outbreak. A major role of the network would be to establish a communication system to connect front-line clinicians and network collaborators at multiple sites to facilitate an exchange of clinical information. This would allow accumulated clinical experience to be shared between sites and would provide a forum for the discussion of patient management issues. Furthermore, it could be used to identify and select hypotheses for evaluation, determine the feasibility of studies, and recruit clinicians to participate in trial design and implementation. The network would shoulder the burden of protocol development and implementation, including liaising directly with local or national ethics boards, thereby reducing workload on the clinicians who will be fully occupied with clinical responsibilities.

## Contingency Fund for Urgent Therapeutic Trials

Trials are resource intensive and cannot be initiated without financial support. Outbreaks are unpredictable events that place enormous strains on healthcare systems. To launch a trial promptly during an outbreak, without diverting local funding away from efforts that are crucial for outbreak control, a centralized contingency fund for urgent clinical therapeutic trials should be established nationally or internationally. Ideally, funding would support an external clinical trials team capable of operationalizing the trial, as local personnel may be overwhelmed by clinical responsibilities related to the outbreak.

## Research Ethics Boards and Ethics Approval

The need to obtain approval from research ethics boards is another factor that may delay initiation of a trial. This requirement is further complicated by the mobile nature of outbreaks. Individual institutions may be affected by an outbreak for limited periods of time; the outbreak may spread to new geographic areas and involve new institutions. Investigators may need to repeatedly submit their protocol to new ethics boards, resulting in further delays and missed opportunities.

Our trial of ribavirin in SARS treatment was approved at several university-affiliated teaching hospitals involved in caring for patients during the first SARS outbreak in Toronto. By the time the trial was approved and ready to start, the outbreak appeared to be over. When a second outbreak occurred, it was centered at a hospital where ethics approval had not been obtained.

Several potential solutions to this problem exist. The most fundamental would be the creation of a national or regional emergency ethics review board with the authority to override the need for local institutional approval and the capability of rapidly assessing protocols when outbreaks occur. In certain circumstances, ethics approval could be obtained prior to an outbreak. For example, a protocol for a trial of corticosteroids in SARS could be designed and approved now and implemented if another outbreak occurs. Additionally, a “rolling” type of ethics approval could be developed, whereby portions of a trial could be approved while other elements are modified in real time as the trial is launched, with ongoing supervision and periodic re-approval by the ethics board. This would allow flexibility in refining dosing regimens and prevent rigid protocols from being repeatedly abandoned as new information emerges.

## Risks to Health Care Workers and Researchers

Transmissibility of pathogens is a feature unique to outbreaks of infectious diseases and has direct implications for conducting clinical trials. In an outbreak, both caregivers and researchers involved in assessing and monitoring patients may be placing themselves at risk. This situation is particularly true in the early stages of an outbreak involving a novel pathogen such as SARS because the most effective means of preventing transmission are not yet known. For example, SARS studies conducted in Toronto required clinicians to obtain specimens such as nasopharyngeal swabs in addition to those collected for clinical purposes. Such actions increase the potential for both exposure to and acquisition of SARS.

In addition to personal risk, researchers may become vectors of disease, placing patients, colleagues, family members, and institutions at risk. Immediately after ethics approval was obtained for our trial, one of the trial’s primary investigators was hospitalized with SARS, and several other researchers were quarantined. Although these exposures did not result from participation in the clinical trial, they highlight the difficulty of conducting clinical trials during an outbreak, especially when researchers are also involved in outbreak management and may be moving between institutions. Evidence that healthcare workers working part-time at several Toronto area hospitals transmitted SARS from one institution to another led to the public health requirement that healthcare workers be limited to a single institution. In particular, healthcare workers at hospitals in which transmission had occurred were not permitted to enter other hospitals. Such policies were important in limiting the spread of the outbreak but provide additional challenges to trial implementation.

In the future, investigators and research ethics boards will need to expand their horizons to consider the risks and benefits of trials as they apply to participating clinicians, researchers, and the public, as well as to the individual patient. In some cases, increasing the potential for exposure of either clinicians or researchers above what is clinically mandated may be considered unethical, particularly when novel pathogens of unknown mode of transmission are involved. Local ethics boards may not have the experience or expertise to deal with issues of public safety; this provides another argument for the creation of a specialized, national ethics board to deal with clinical trials during outbreaks or other public health emergencies.

Various approaches could be utilized to manage these risks. A cautious approach to preventing transmission and providing adequate training for participating clinicians and researchers should be required. Integrating clinical care and trial monitoring can reduce the number of patient visits. Sharing specimens between clinical and research laboratories may eliminate the need for additional specimen collections, and symptom assessment by telephone interview would minimize the need for patient contact. The study should be coordinated centrally and communication between study sites should be by teleconference and email, until risks are defined and minimized. Travel between centers would be limited by providing data collection forms designed to be directly scanned and transmitted via the Internet. This would reduce transmission risk and also allow the participation of multiple international sites in a single trial. Similar approaches have been effective in other areas of clinical research and can also increase study enrollment (*18*).

## Conclusions

Clinicians caring for patients with infectious disease syndromes caused by unknown or novel pathogens are under intense pressure to offer potentially efficacious therapies. In future outbreaks, as in the SARS outbreak, therapeutic agents of unknown benefit will likely be used again. These agents should be used in the setting of trials, to determine their efficacy and prevent therapies of unknown efficacy (e.g., corticosteroids for SARS) from becoming “standard of care” in the absence of good evidence.

The unpredictable nature of outbreaks poses many challenges to the successful design and implementation of such trials. Creation of national or international collaborative groups, with a mandate to implement clinical trials of therapeutic agents in outbreak settings, and supported by appropriate funding, may be the best strategy for achieving this goal. The collaboration of ethics review boards in establishing a process which facilitates trials while ensuring the safety of participating patients, researchers, and communities, is critical.

Following the SARS outbreak in 2003, thousands of patients were treated with agents of unproven efficacy and definite toxicity without the accumulation of good data on their efficacy. To prevent this situation from repeating itself, we must be prepared to conduct prospective, controlled trials in the event of future outbreaks of novel pathogens.
